# Pituitary abscess: a descriptive analysis of a series of 19 patients—a multi-center experience

**DOI:** 10.1186/s40001-024-01749-z

**Published:** 2024-05-02

**Authors:** Qiang Xue, Xiuhua Shi, Xiaoling Fu, Yating Yin, Hui Zhou, Suiyi Liu, Qingfang Sun, Jin Meng, Liuguan Bian, Hong Tan, Hua He

**Affiliations:** 1 Departments of Neurosurgery, The Third Affiliated Hospital, Naval Medical University, Shanghai, 200438 China; 2 Department of Radiotherapy & Oncology, The No. 2 People’s Hospital of Wuhu City, Wuhu, Anhui China; 3 Department of Medical Psychology, The Fourth Medical Center of PLA General Hospital, 51 Fu Cheng Road, Beijing, 100048 China; 4 Department of Radiology, Xiangya Hospital, Central South University, Changsha, China; 5 Department of Medical Engineering, Third Affiliated Hospital of Navy Military Medical University, Shanghai, 200438 China; 6 Department of Neurosurgery, Ruijin Hospital, Shanghai JiaoTong University School of Medicine, Rui Jin Er Road, Shanghai, 200025 China; 7 Institute of Organ Transplantation, Changzheng Hospital, Navy Medical University, Shanghai, China; 8 Department of Anesthesiology, Huashan Hospital, Fudan University, Shanghai, China

**Keywords:** PA, Diagnosis, Treatment, Transsphenoidal surgery

## Abstract

**Objectives:**

Pituitary abscess (PA) accounts for only 0.3–0.5% of sellar masses, and the lack of specific clinical symptoms makes diagnosing PA difficult without a surgical biopsy. In clinical practice, PA is often mistaken for cystic pituitary adenoma, craniopharyngioma, and Rathke’s cyst. Thus, this study aims to investigate challenges in diagnosing PA and evaluate the importance of combining intraoperative surgery with postoperative antibiotic treatment.

**Methods:**

We conducted a retrospective analysis of 19 patients diagnosed with PA through histopathology. All patients underwent transsphenoidal surgery (TSS) for pituitary adenomas after undergoing comprehensive preoperative evaluations, including routine tests, endocrine assay, and imaging examination. Furthermore, we compared different treatments for pituitary abscess (PA) to determine the most effective approach for achieving a favorable prognosis.

**Results:**

The most prevalent symptom of PA was headache, especially in the frontal–temporal and vertex regions, ranging from mild to moderate severity. Hypopituitarism-related symptoms were also frequently observed, including hypaphrodisia, cold sensitivity, fatigue, weight loss, polyuria, and amenorrhea. Twelve patients exhibited abnormalities in endocrinology examinations. Diagnosing PA correctly is challenging. In our study, none of the patients were correctly diagnosed with PA prior to surgery, and many sellar lesions were misdiagnosed. The favorable prognosis was largely attributed to surgical intervention and active postoperative antibiotic therapy.

**Conclusions:**

Given the lack of clarity in preoperative diagnosis, typical intraoperative findings and effective antibiotics treatment are more indicative of the correct diagnosis than other tests. In terms of therapy, optimal surgical intervention and active postoperative antibiotic treatment contribute to resolving the challenges posed by PA.

## Introduction

Pituitary abscess (PA) accounts for only 0.3–0.5% of sellar masses, and the epidemiological studies suggest that it accounts for less than 0.2–1% of all pituitary lesions [1, 2]. PA can be classified into primary and secondary forms. Primary PA occurs in normal pituitary tissue and is typically caused by the spread of local infections, such as sphenoid sinusitis, meningitis, intracranial infection caused by cerebrospinal fluid leakage, cavernous sinus thrombosis, or infection following transnasal sphenoid surgery. Secondary PA refers to an abscess following pituitary lesions, such as pituitary adenoma, Rathke's cyst, or craniopharyngioma. Approximately one-third of secondary PA patients with pituitary disorders were reported in the literature [3].

Common clinical manifestations of PA include headaches, visual disturbances, hypopituitarism, and meningeal irritation resulting from abscess compression of surrounding structures [4]. Nevertheless, the absence of specific clinical symptoms makes diagnosing PA difficult without a surgical biopsy.

Combining the imaging features of computed tomography (CT) scanning and magnetic resonance imaging (MRI) in the anterior skull base and diaphragm sellae with clinical manifestations [5, 6] has been suggested as an effective non-invasive method for the diagnosis of PA. In the meantime, another researcher has demonstrated that symptoms such as meningitis, sinusitis, sepsis, and rapid neurological deterioration could be indicative of abscess formation due to pituitary adenoma [7]. Moreover, Anagnos emphasized the importance of diabetes insipidus (DI) as a characteristic symptom for distinguishing adenoma from PA in patients [8]. In clinical practice, the PA is frequently confused with cystic pituitary adenoma, craniopharyngioma, and Rathke’s cyst.

In the present study, we sought to classify the clinical characteristics of PA by analyzing a cohort of 19 patients from three medical centers, providing clinicians with a diagnostic reference for PA. Furthermore, we compared various treatments for PA to identify the most efficacious method for achieving a favorable prognosis.

## Materials and methods

We retrospectively reviewed 19 patients diagnosed with PA from Shanghai Ruijin Hospital, Shanghai Changzheng Hospital, and Hunan Xiangya Hospital between May 2017 and November 2022. Inclusive criteria include (1) Patients diagnosed with PA with histological evidence of acute or chronic inflammation and abscess wall; (2) Patients who completed the whole treatment process in our center. Exclusion criteria are as follows: (1) conservative patients who could not be diagnosed with PA; (2) patients who died during hospitalization due to other diseases; and (3) patients who lost follow-up after discharge. After enrolling, we documented their presenting symptoms, including fever, typical imaging findings, endocrine observations, and drainage of pus-containing organisms. Each patient underwent a comprehensive medical history review, followed by thorough neurological and ophthalmologic assessments. Preoperatively and postoperatively (from 3 months to 9 years), contrast-enhanced MRI scans were performed, with concurrent monitoring of baseline and postoperative endocrine functions over time. All 19 patients underwent transsphenoidal surgery for abscess evacuation. In addition, all patients received both oral and intravenous antibiotics. The earliest patients to use antibiotics were taken 3 days before surgery, while most patients taking them after surgery, with a time span from 3 days before surgery to 4 weeks after surgery. Hormone replacement therapy was given to patients that suffered from hypopituitarism. All our centers routinely performed follow-ups through outpatient visits and telephone interviews.

## Results

### General characteristics

The mean age of the 19 patients in this cohort was 43.4 years (ranging from 16 to 77 years), with 12 females and 7 males. The mean follow-up period was 45.3 months (ranging from 2 to 112 months). All patients had no history of radiotherapy or chemotherapy, immunosuppressant application, AIDS, alcohol or drug abuse. None of these patients had undergone previous surgery for pituitary or sphenoid sinus lesions. Five (26%) had a history of possible infection: two with submandibular lymphnoditis, one with sphenoid sinusitis, and another two with previous cholecystitis (Table 1).

**Table 1:** General information and clinical presentations

Case No	Sex and age	Complaints	Signs symptoms	Vision inspection	Previous history	Surgical method	Follow up (m)
1	32 F	Amenorrhea	Afebrile; nl WBC	VF normal	None	TSS	36
2	58 F	HA	Afebrile; nl WBC	VF normal, VS abnormal	Cholecystitis	TSS	30
3	60 F	HA, Nausea, Vomiting	Afebrile; nl WBC	VF normal	None	TSS	48
4	77 M	HA, Nausea, Vomiting	Febrile; nl WBC	VF normal	T2DM, Colon cancer	TSS	2
5	52 M	HA	Afebrile; nl WBC	VF abnormal	None	TSS	72
6	36 M	Sexual dysfunction, Myasthenia	Afebrile; Evaluated WBC	VF normal	None	TSS	34
7	49 F	Dizzy, Feeble, Hypermelanosis	Afebrile; nl WBC	VF normal	Sphenoid sinusitis	TSS	18
8	38 F	Amenorrhea	Afebrile; Evaluated WBC	VF normal	Submandibular lymphnoditis	TSS	70
9	64 M	Nausea, Vomiting Drowsiness	Febrile; Evaluated WBC	VF normal	Hypertension	TSS	2
10	53 F	HA	Afebrile; nl WBC	VF normal, VS abnormal	Cholecystitis	TSS	108
11	31 M	Neg	Afebrile; nl WBC	VF normal	None	TSS	12
12	16 M	Neg	Afebrile; nl WBC	VF normal	None	TSS	8
13	49 F	HA	Afebrile; nl WBC	VF normal	None	TSS	14
14	29 F	HA	Afebrile; nl WBC	VF normal VS abnormal	None	TSS	76
15	24 M	HA	Febrile; Evaluated WBC	VF normal	Submandibular lymphnoditis	TSS	68
16	68 F	HA	Afebrile; nl WBC	VF normal	None	TSS	83
17	24 F	HA	Afebrile; nl WBC	VF normal	None	TSS	112
18	40 F	Dizzy, Amenorrhea, Feeble, Polydipsia Polyuria	Afebrile; nl WBC	VF normal	None	TSS	42
19	24 F	HA, Amenorrhea,	Afebrile; nl WBC	VF abnormal	None	TSS	26

No (number); yrs (years); M (male); F (female); Neg (negative); WBC (white blood cell); HA (headache); nl (normal level) m (month); MRI (Magnetic Resonance Imaging); VF (vision field); VS (vision sensitivity); A.M. (ante meridiem); P.M. (post meridiem); none (not found)

The most common symptom in our series was headache, affecting a substantial proportion (11, 58%) of patients. Most complained of long-standing headaches in the frontal–temporal and vertex areas ranging from mild to moderate degrees. In contrast, only one patient experienced a severe headache at the onset. Hypopituitarism-related symptoms were also common (6, 32%) at presentation, such as hypaphrodisia, cold sensitivity, fatigue, weight loss, polyuria, and amenorrhea. Five patients (26%) suffered from visual disturbances, either unilateral or bilateral changes in their visual fields, visual acuity or double vision. Four patients (21%) presented with elevated peripheral white blood cell (WBC) counts, and three (16%) presented with febrile. Three patients (16%) experienced nausea and vomiting, including two presenting with febrile and one with elevated peripheral WBC counts and drowsiness. One patient suffered from polyuria and polydipsia, with polyuria occurring more than ten times per day, and drinking more than 1.5-L water daily.

### Endocrinology assays

Twelve patients were found to have abnormalities following endocrinology examinations conducted on all patients. Endocrinology tests revealed potential deficiencies in thyroid, adrenal cortical, and gonad function (Table 2).

**Table 2:** Preoperative endocrinology hormones level

Case No	Endocrine findings	Thyroid hormone	Adrenocortical hormone	Gonadal hormone	Growth hormone (GH)	Adrenocorticotrophic hormone (ACTH)	Prolactin (PRL)
1	Abnormal	T3:0.72; FT3:1.97	BFC:26.40-25.30-25.80, UFC:1026.10	FSH:0.61, LH:0.13	1.26	22.80	12.76
2	Normal	T3:1.67; T4:134.52 FT3:3.80; sTSH:0.73	BFC:10.2-6.4-10.80, UFC:74.10	FSH:36.93 LH:8.26,	1.08	45	13.74
3	Normal	T3:1.57, T4:135.03, FT3:3.81, FT4:14.49, TSH:2.99	BFC:8.8-18.3-1.90, UFC:953.20	FSH:5.4, LH:9.62	2.24	53.10	11.64
4	Abnormal;	T3:0.90, T4:57.08, FT3:2.74, FT4:10.41, sTSH:0.18	BFC:2.81-1.09-19.09, UFC:664.40	FSH:7.21, LH:1.44	0.47	16.80	5.61
5	Normal	T3:1.59, T4:105.39, FT3:4.98, FT4:14.35, sTSH:1.25	BFC:9.03-12.6-11.4, UFC:85.60	FSH:5.72, LH:2.47	1.3	39	12.1
6	Abnormal	T3:3.01, T4: < 5.15, TSH:0.96	BFC:5.3-14.2-7.9, UFC:23	FSH:4.9, LH:7.2	1.3	33	31.74
7	Abnormal	T3:1.51, T4:37.12, FT3:3.63, FT4:9.34, sTSH:0.127	BFC:2.10-2.90-1.70, UFC:6.50	FSH:1.71, LH:0.12	0.12	19	74.96
8	Abnormal	FT3:3.08, FT4:12.23, sTSH:1.92	BFC:8.8-3.3-5.2; UFC:67.7	FSH:2.08, LH:0.76	1.74	28	11.79
9	Abnormal	T3:1.88; T4:126.8 FT3:4.48 FT4:15.6 sTSH:0.01	BFC:2.2-22-17, UFC:420	FSH:0.61, LH:0.13	2. 6	41.90	35.70
10	Abnormal	T3:1.88; T4:126.8 FT3:4.48 FT4:15.6 sTSH:0.01	BFC:2.2-22-17, UFC:420	FSH:0.61, LH:0.13	2. 6	41.90	35.70
11	Normal	T3:0.65, T4:35.17, FT1.97, FT4: 4.44, TSH:1.13	BFC:25.85-18.3-1.90, UFC:953.20	FSH:5.4, LH:9.62	1.8	24.30	4.76
12	Normal	T3:1.10, T4:27.78, FT3:2.42, FT4:4.31, sTSH:6.47	BFC:95-1.09-19.09, UFC:664.40	FSH:7.21, LH:1.44	1	46.90	5.61
13	Normal	T3:1.08, T4:82.98, FT3:2.90, FT4:11 sTSH:3.82	BFC: 204-192-102, UFC:85.60	FSH:51.0, LH:23.48	1.8	25.20	17.92
14	Abnormal	T3:3.01, T4: < 5.15, TSH:0.96	BFC:5.3-11.2-9.5 UFC:23	FSH:4.9, LH:7.2	1.3	33	31.74
15	Abnormal	T3:1.51, T4:37.12, FT3:3.63, FT4:9.34, sTSH:0.127	BFC:2.10-2.90-1.70, UFC:6.50	FSH:1.71, LH:0.12	0.12	19	74.96
16	Abnormal	FT3:3.08, FT4:12.23, sTSH:1.92	BFC:8.8-3.3-5.2; UFC:67.7	FSH:2.08, LH:0.76	1.74	28	11.79
17	Abnormal	FT3:37.74, FT4:77.73, sTSH: < 0.005	Untested	FSH:14.89 LH: < 0.10	1.72	Untested	1.13
18	Normal	FT3:3.1, FT4:24, TSH:0.1	Untested	FSH:7.01, LH: < 2.53	Untested	Untested	47.25
19	Abnormal	T3:1.35, T4:78.3, TSH:2.44	Untested	FSH:0.53, LH: < 0.54	0.66	Untested	67.82

T3: triiodothyronine, T4: tetraiodothyronine, FT3:free triiodothyronine, FT4: free tetraiodothyronine, TSH: thyroid stimulating hormone, sTSH: sensitive thyroid stimulating hormone, BFC: blood free cortisol, UFC: urine free cortisol, FSH: follicle stimulating hormone, LH: luteinizing hormone

### Imaging studies

**Fig. 1:**
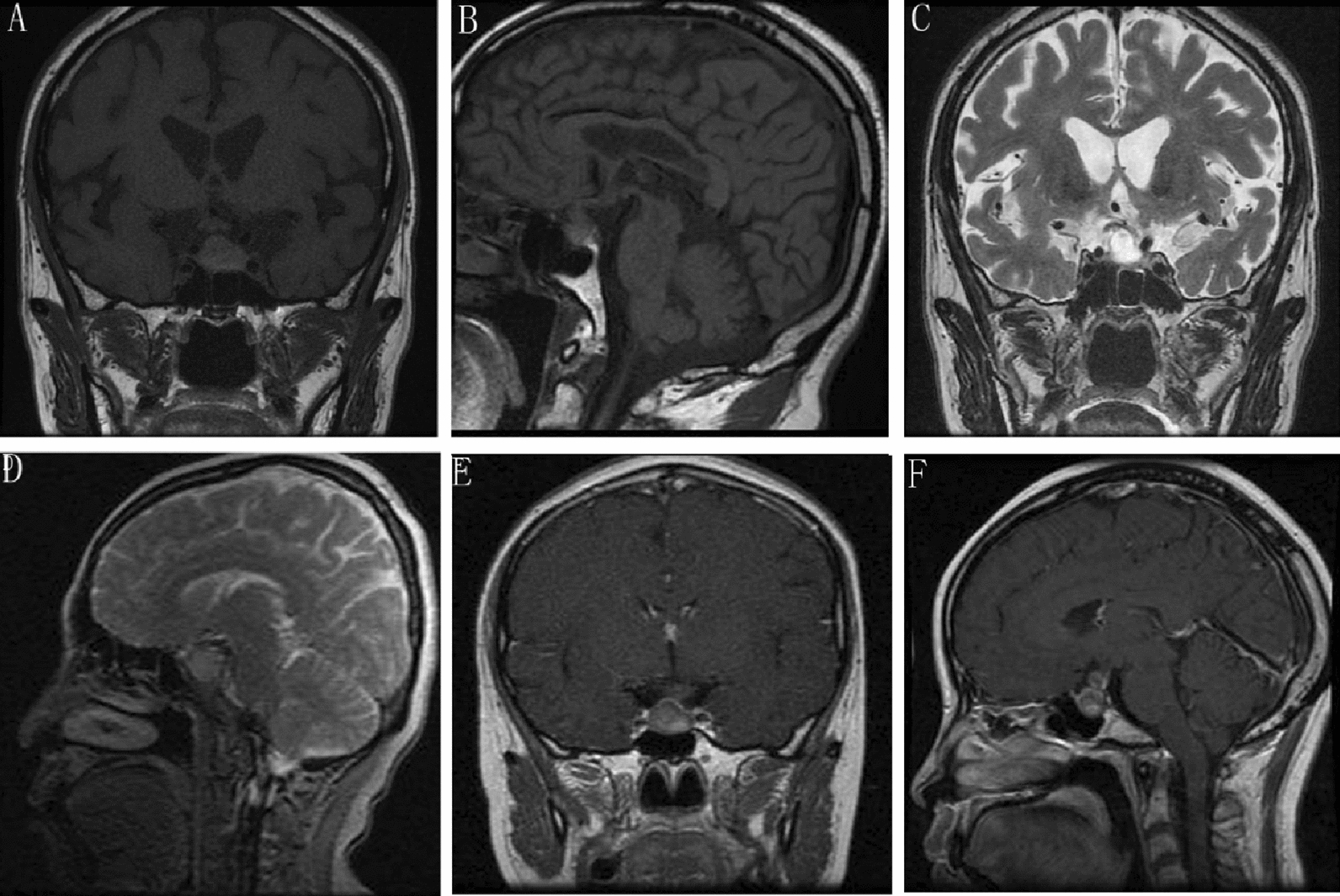
MRI of PA preoperative: **A** T1WI coronal: iso-hyperintense signal; **B** T1WI sagittal: iso-hyperintense signal; **C** T2WI coronal: hyperintense signal revealed a sellar heterogeneous; **D** T2WI sagittal: hyperintense signal; **E** T1WI coronal contrast-enhanced: heterogeneous hyperintense signal, lesion with rim enhancement; **F** T1WI sagittal contrast-enhanced: heterogeneous hyperintense signal, lesion with enhancement

Sellar MRI is often considered the most valuable and indicative diagnostic tool for identifying sellar lesions. MRI can reveal enlargement of the sellar turcica. Many of these abscesses exhibited moderate to high signal intensity on T2-weighted images (T2WI), indicative of a fluid-containing cyst. T1-weighted images (T1WI) demonstrated that the masses, when compared with the surrounding brain, tended to exhibit signal characteristics ranging from hypointense to mildly hyperintense, while T2WI demonstrated peripheral or rim enhancement. In two cases, "waist" and "gourd" shapes were also present. Fourteen patients (74%) exhibited a hypo-intense signal of sellar mass on T1WI, with two (10%) showing a hyper-intense signal and three (16%) showing an iso-intense signal. Nine cases (47%) demonstrated a hyperintense sellar mass with the peripheral contrast-enhancing rim on T1WI (due to increased protein content), while ten (53%) exhibited significantly abnormal, uneven enhancement of the pituitary (Fig. 1; Table 3).

**Table 3:** Preoperative MRI imaging and vision inspection

Case No	Imaging modality findings	T1WI	T2WI	Contrast injection
1	MRI	T1:hyper	T2:hyper	Optic chiasm compressed
2	MRI	T1:hypo	T2:hyper	Enh: rim mild
3	MRI	T1:hypo	T2:hyper	Enh: rim mild; stalk thicker
4	MRI	T1:hypo	T2:hyper	Enh: Peripheral rim
5	MRI	T1:hypo	T2:hyper	Enh: mild
6	MRI	T1:hypo	T2:hyper	Enh: mild
7	MRI	T1:hypo	T2:hyper	Enh: mild
8	MRI	T1:iso	T2:hyper	Enh: mild
9	MRI	T1:iso	T2:hyper	Enh: mild
10	MRI	T1:hypo	T2:hyper	Enh: rim Enhanced
11	MRI	T1:hypo	T2:hyper	Enh: rim mild; stalk thicker
12	MRI	T1:hypo	T2:hyper	Enh: Peripheral rim
13	MRI	T1:hypo	T2:hyper	Enh: rim Enhanced
14	MRI	T1:hypo	T2:hyper	Enh: mild
15	MRI	T1:hypo	T2:hyper	Enh: mild
16	MRI	T1:iso	T2:hyper	Enh: mild
17	MRI	T1:hypo	T2:hyper	Enh: rim mild
18	MRI	T1:iso-hyper	T2:hyper	Enh: Peripheral rim
19	MRI	T1:hypo	T2:hyper	Enh: mild

MRI (magnetic resonance imaging); iso (iso-signal); hyper (hyper-signal); hypo (hypo-signal); Enh: Enhanced

### Preoperative diagnosis

None were accurately diagnosed with PA prior to surgery. Twelve (63%) patients were preoperatively diagnosed as pituitary adenoma, two (10%) as pituitary adenoma with apoplexy, and the remaining (27%) patients were misdiagnosed as Rathke's cyst.

### Intraoperative exploration and management

Various mixtures were observed, including creamy or white-green pus, and viscous or jelly-like substances. The intrasellar cystic mass of capsule specimens was completely excised, with volumes ranging from 1 to 9 mL. To prevent infection, the sellar was irrigated with a large amount of saline, gentamicin, or povidone-iodine and packed with a small lump of abdominal fat, guided by a provisional diagnosis of PA. The anterior wall of the sellar was reconstructed using vomer bone, sealed with fibrin glue to prevent and minimize intraoperative and postoperative complications.

### Pathological examination

**Fig. 2:**
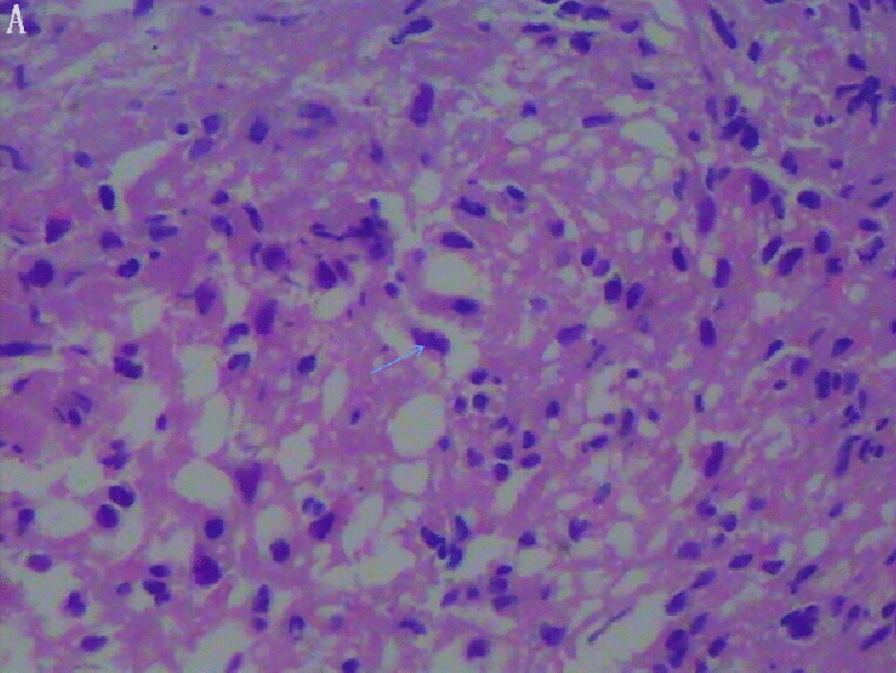
Reveals a lymphocyte-infiltrated abscess wall; the arrow indicates a foamy histiocyte (hematoxylin–eosin stain)

The intraoperative frozen section revealed features of PA, including an abscess wall rich in foamy histiocytes (Fig. 2), as well as pituitary tissue degeneration and necrosis with accompanying inflammatory cell infiltration. In contrast, the paraffin section confirmed the diagnosis of PA. A histological examination of pus and the abscess wall confirmed the presence of inflammatory cells. Only one of the Gram-stained or cultured samples tested positive for staphylococcus, while the others tested negative (Table 4).

**Table 4:** Pathology and postoperative treatment

Case No	Pus volume (mL)	Gram stain/cultures	Antibiotic Rx	Endocrine treatment	Outcome
1	9	Neg	Sulbactam and Cefoprazone	Hydrocortisone acetate replacement	Recovery
2	5–8	Neg	Lincomycin	Hydrocortisone acetate replacement	HA improved
3	1.5	Staphylococcus positive	Sulbactam and Cefoprazone	Hydrocortisone acetate replacement	HA improved
4	4–7	Neg	Ceftriaxone	Hydrocortisone acetate replacement	HA improved
5	5–6	Neg	Ceftriaxone	Hydrocortisone acetate replacement	HA improved
6	1–1.5	Neg	Cefotiam	Hydrocortisone acetate replacement	Recovery
7	1–1.5	Neg	Ceftriaxone	Hydrocortisone acetate replacement	Recovery
8	7–8	Neg	Ceftriaxone	Hydrocortisone acetate replacement	Recurred 6 years later and accepted the second surgery
9	1.8	Neg	Vancomycin and Metronidazole	Hydrocortisone acetate replacement	Recovery
10	2	Neg	Vancomycin	Hydrocortisone acetate replacement	Recovery
11	3	Neg	Vancomycin and Metronidazole	Hydrocortisone acetate replacement	Recovery
12	3–5	Neg	Vancomycin and Ornidazole	Hydrocortisone acetate replacement	Recurred 5 months later and accepted the second surgery
13	4.3	Neg	Cefotiam	Hydrocortisone acetate replacement	Recovery
14	3	Neg	Vancomycin and Metronidazole	Hydrocortisone acetate replacement	Recovery
15	2.5	Neg	Vancomycin and Metronidazole	Hydrocortisone acetate replacement	Recovery
16	3.2	Neg	Vancomycin and Metronidazole	Hydrocortisone acetate replacement	Recovery
17	2	Neg	Ceftriaxone	Hydrocortisone acetate replacement	Recovery
18	5	Neg	Ceftriaxone	Hydrocortisone acetate replacement	Recovery
19	8	Neg	Ceftriaxone	Hydrocortisone acetate replacement	HA improved

No: number, Neg: negative, HA: headache

### Medical treatment

Antibiotics are typically administered for more than 2 weeks, while endocrine replacement therapy for hypopituitarism related to pituitary function usually lasts for 1–2 weeks. In our clinical practice, suspected pituitary abscesses may be treated empirically with oral antibiotics to observe changes in pituitary imaging. Upon diagnosis of pituitary abscess, intravenous antibiotics are the primary treatment choice, supplemented by oral antibiotics and nasal topo-anti-infective. Monitor pituitary function by adjusting levels of hydrocortisone acetate before and after surgery. For instance, 100 mg of succinate hydrocortisone was initially administered intravenously for about 3 days, followed by a reduction to 25 mg taken orally twice daily at 8 ante meridiem(AM) and 4 post meridian(PM), respectively, and subsequently decreased to 25 mg once daily at 8 AM. Additional supportive postoperative care, including hemostasis, gastric mucosal protection, management of diabetes insipidus and potassium abnormalities, and emotional support, is also necessary.

### Follow-up and prognosis

Follow-up periods in our cohort ranged from 2–112 weeks. All patients with preoperative headaches experienced significant symptom improvement, as did the five patients with visual disturbances. Two patients experienced recurrence 6 years and 5 months apart, necessitating a second operation. Currently, all patients have fully resumed normal activities.

## Discussion

### Epidemiology and etiology

Since Simmonds reported the first case of PA in 1914, over 210 cases have been documented in the medical literature [9, 10]. PA is most prevalent between the ages of 3 and 69, with a clinical incidence rate ranging from 0.3% to 0.5% of sellar masses, irrespective of age or gender [11, 12]. According to our research, it represented 0.4% of all lesions in the sellar region. In terms of etiology, PA can be classified into primary and secondary PA based on the pathogenic organism involved. Primary PA occurs in normal pituitary tissue and is typically caused by the spread of local infection.

In contrast, secondary PA is an abscess that develops after pituitary lesions such as pituitary adenoma, Rathke's cyst, craniopharyngioma, or sellar surgery. [13–15]. The source of infection cannot be identified in more than 60% of PA cases, which may be attributed to cryptogenic causes [16]. In our cohort, fourteen patients (74%) showed no evidence of infection, while two had submandibular lymphnoditis, one had sphenoid sinusitis, and two had a history of cholecystitis.

### Clinical manifestations

Headache (91.7%) and visual disturbances (58%) are the most common symptoms, according to the literature [17, 18]. Danilowicz reported that 75–100% of patients with visual impairment and visual field defects, 54.2% of patients had anterior pituitary dysfunction, resulting in diverse clinical manifestations such as adrenocortical adenoma, adrenocortical adenoma, adrenocortical ade decreased libido, cold intolerance, amenorrhea, polyuria, and polydipsia, and it may lead to panhypopituitarism [19]. DI almost accounts for nearly 50% of PA compared to 10% of pituitary adenoma, suggesting that DI can be helpful in diagnosing PA [20]. In addition, approximately 33.3% of patients presented with fever, 33.3% had an elevated white blood cell count, and 25% had meningismus [21]. In our cohort, six patients (32%) exhibited symptoms related to hypopituitarism, including hypaphrodisia, cold sensitivity, fatigue, weight loss, polyuria, and amenorrhea, five patients (26%) experienced visual disturbances, four patients (21%) had elevated peripheral WBC counts, and three patients(16%) presented with febrile illness (Table 5). In addition, three patients (16%) experienced nausea and vomiting, while only one exhibited symptoms of polyuria and polydipsia. Sometimes, cases lacking inflammatory or meningeal symptoms may be associated with mass abscess effect [22, 23]. Studies have revealed that pituitary hormone deficiency is typical among PA patients, as the abscess can obstruct the hypothalamus or pituitary stalk release [24]. Dutta found that pituitary stalk lesions block the production of PRL release inhibitors, resulting in an increased PRL level [25]. Reduced libido, polyuria, polydipsia, cold intolerance, and amenorrhea are the most common indications of impaired endocrine function. Growth hormone deficiency is reported as the earliest symptom, followed by deficiencies in follicle-stimulating hormone/luteinizing hormone, thyroid-stimulating hormone, and adrenocorticotrophic hormone [26].

**Table 5:** Incidence of clinical manifestations

	Hypopituitarism-related symptoms (%)	Visual disturbances (%)	Elevated peripheral WBC counts (%)	Febrile illness (%)	Nausea and vomiting (%)
Vates, G [42]	54.2	75–100	33.3	33.3	–
Liu, F [39]	84.8	27.3	18.2	21.2	18.2
Our research	32	26	21	16	16

### Pathogenesis

The direct spread or hematogenous dissemination of sphenoid sinusitis may play a role in the pathogenesis, which is the most commonly reported mechanism of abscess formation [27]. Kroppenstedt reported a close association between PA formation and blood circulation disorders, as well as tumor necrosis resulting from systemic or regional immune dysfunction [28]. Some believe that a sterile abscess may result from invasive pituitary necrosis, liquefaction of atypical pituitary cyst contents, or an atypical pituitary cyst [29]. Others argue that it may be attributed to inadequate bacteriological technique or antibiotic therapy before or during surgery [30]. Positive results for microorganisms are occasionally observed in PA, with gram-positive organisms, typically cocci, such as *Staphylococcus* or *Streptococcus* species, being the most common. Gram-negative or coliform bacteria, such as *Neisseria, E. coli*, and *Corynebacteria*, as well as uncommon organisms like fungi and parasites, constituted a small proportion [31]. Histopathological examination of PA specimens often reveals several inflammatory cells, including neutrophils, lymphocytes, and plasma cells, which could simulate the infection process and accompany the abscess formation [32].

### Radiographic studies

Imaging is a valuable diagnostic tool for PA. CT scans and skull radiographs are nonspecific. Skull radiographs may reveal enlargement of the sella, erosion of the sellar floor, and opacity of the sphenoid sinus. In contrast, CT scans typically reveal sellar enlargement and a low-density sellar mass with contrast enhancement [33]. Similar to pituitary adenoma, erosion and expansion are the most common findings in the sella. Diffusion-weighted imaging (DWI) may be useful for distinguishing among PA, apoplexy, and pituitary adenoma. MRI is the most effective method for the preoperative diagnosis of PA. On MRI, this mass shows typical abscess characteristics, such as a cystic or partially cystic lesion in the sellar region. It appears as a low signal on T1WI and a high signal on T2WI. After gadolinium injection, there is accentuation of the lesion's periphery, and the signal enhancement is attributed to the presence of protein. The peripheral rim enhancement is believed to be suggestive of PA, such as hyperintense ring enhancement on T1WI that may be caused by hemorrhage and necrosis on liquid with high protein content [34]. Local thickening of the pituitary stalk may also suggest the presence of PA [35]. In cases of suspected PA, hyperintense and hypointense signals from the abscess wall are more indicative.

### Diagnostic quandary

Diagnosing PA is challenging and requires a comprehensive medical history, physical examination, and a high index of suspicion. Unfortunately, specific preoperative diagnostic procedures for PA are lacking. As mentioned previously, a history of meningitis, sinusitis, or sepsis may suggest the presence of PA. Rapid neurological deterioration in a patient with a sellar tumor following suspected bacteremia should raise suspicion of abscess formation. According to the literature, suspicion of PA should be raised in the presence of the following: a history of associated diseases or an unexplained elevation in blood WBC; early hypopituitarism symptoms such as DI in the absence of a saddle tumor. Widening of the sellar turcica, erosion of the sellar floor and bone, and opacity of the sphenoid sinus on skull radiograph, CT, and MRI are indicative of a peripheral ring or sellar lesion with homogeneous enhancement [36, 37]. When assessing a patient with symptoms of hypopituitarism and a pituitary cystic mass showing heterogeneous intensity on imaging, suspicion of infection is warranted if the patient exhibits DI and amenorrhea or symptoms and signs of pituitary mass and infection. Differential diagnoses for sellar lesions include adenoma, carcinoma, arachnoids' cyst, colloid cyst, Rathke's cyst, craniopharyngioma, and metastasis. Many of these tumorous and non-tumorous lesions may mimic the clinical, endocrine, and radiographic manifestations of pituitary adenomas, complicating the differentiation between these potential etiologies. A skilled surgeon can distinguish intraoperatively between pituitary necrosis and Rathke's cyst [38].

### Treatment recommendations and outcomes

Surgical drainage followed by antibiotic treatment is the recommended therapy for PA [3]. With appropriate management, the prognosis for PA is generally favorable. The transsphenoidal approach is preferred over craniotomy due to lower risks of infection spread and visual impairment, and 75% of PA patients experience complete resolution of visual abnormalities following TSS. All patients underwent surgery via endoscopic endonasal transsphenoidal approach. Although surgery cannot completely remove the abscess, it prevents its spread to the subarachnoid space, reduces postoperative infection rate and mortality, and shortens operation time and hospital days [39]. The mortality rate has been reduced to 8.3% by early diagnosis and aggressive antibiotic treatment [1]. If there is a strong suspicion of the diagnosis, empirical antibiotics should be promptly initiated, adjusted after identifying the focus, and continued for 2–6 weeks. In our clinical practice, we would like to use gentamicin to flush rather than vancomycin during our surgery. Ceftriaxone can be used for empirical treatment first, and then replaced with sensitive antibiotics when microbiological and histological evidence is available. Antibiotics were stopped after complete resolution of the infection. Ciappetta used TSS to eliminate the abscess, triiodomethane gauze to drain pus, and antibiotics for 3 weeks postoperatively to achieve a favorable outcome [40]. According to the literature, more than 60% of patients fully recovered, 30% showed improvement in hormonal or visual impairments, and approximately 10% died after surgical and medical treatment [41]^.^ Vates reported that approximately 54.2% of patients suffered from endocrine dysfunction before surgery, of whom 38.5% did not improve postoperatively without developing a new pituitary dysfunction [42]. In comparison, two of our patients experienced a recurrence. The recurrence rate in our cohort was not particularly low. The recurrence rate in the Vates series was 18.8%, in the Liu series was 13.3%, and other studies have reported similar rates [39–42]. Some may considered that the majority of recurrences were in middle-aged female patients with immunological system disorders or with previous surgery, however, further research is needed.

The prognosis is favorable with appropriate treatment, including surgery, antibiotics, and hormone replacement therapy. Complete eradication of PA is the primary objective. All pus should be thoroughly drained during the procedure, and the abscess should be irrigated with gentamicin and saline solution repeatedly. It is essential to protect normal pituitary tissue and prevent damage to the sellar diaphragm to prevent cerebrospinal fluid rhinorrhea and intracranial infection. Evaluate the abscess cavity if a pituitary adenoma is present [8]. Pus collected during surgery must undergo routine microbiological analysis. Ciappetta reported that the combined mortality rate of PA and meningitis was 28%, which rose to 50% when combined with a large sellar tumor [43]. Among these patients, 12.5% died due to new endocrine dysfunction associated with their PA, 62.5% experienced persistent pituitary dysfunction necessitating long-term hormone replacement therapy, and 25% fully regained their endocrine function.

## Conclusion

PA is a rare but potentially life-threatening condition. Key aspects of managing PA include early diagnosis through effective TSS, rational antibiotic therapy, appropriate symptomatic treatment, and long-term follow-up. To prevent the spread of infection and the recurrence of abscesses, high-dose antibiotic therapy is necessary both before and after surgery. Although several signs and symptoms may indicate PA, it is necessary to look for additional indicators to prompt preoperative diagnosis and comprehensive treatment.

## Data Availability

The data sets generated for this study are available on request to the corresponding author.

## Abbreviations

PA: Pituitary abscess
TSS: Transsphenoidal surgery
CT: Computed tomography
MRI: Magnetic resonance imaging
y: Year
m: Month
L: Liter
T1WI: T1-weighted images
T2WI: T2-weighted images
d: Day
w: Week
AM: Ante meridiem
PM: Post meridian
DI: Diabetes insipidus
